# Occupational stressors and work accidents among health workers

**DOI:** 10.11606/s1518-8787.2021055002938

**Published:** 2021-12-02

**Authors:** Mariana Rabelo Gomes, Tânia Maria de Araújo, Jorgana Fernanda de Souza Soares, Camila Carvalho de Sousa, Iracema Lua

**Affiliations:** I Universidade Federal da Bahia Instituto de Saúde Coletiva Programa de Pós-Graduação em Saúde Coletiva Salvador BA Brasil Universidade Federal da Bahia. Instituto de Saúde Coletiva. Programa de Pós-Graduação em Saúde Coletiva. Salvador, BA, Brasil; II Universidade Estadual de Feira de Santana Departamento de Saúde Feira de Santana BA Brasil Universidade Estadual de Feira de Santana. Departamento de Saúde. Feira de Santana, BA, Brasil; III Universidade Federal da Bahia Faculdade de Medicina da Bahia Programa de Pós-Graduação em Saúde, Ambiente e Trabalho Salvador BA Brasil Universidade Federal da Bahia. Faculdade de Medicina da Bahia. Programa de Pós-Graduação em Saúde, Ambiente e Trabalho. Salvador, BA, Brasil; IV Universidade Estadual de Feira de Santana Programa de Pós-Graduação em Saúde Coletiva Feira de Santana BA Brasil Universidade Estadual de Feira de Santana. Programa de Pós-Graduação em Saúde Coletiva. Feira de Santana, BA, Brasil

**Keywords:** Health Personnel, psychology, Accidents, Occupational, prevention & control, Occupational Exposure, Containment of Biohazards, Inservice Training

## Abstract

**OBJECTIVE:**

To test the association between occupational stressors and work accidents due to exposure to biological material (ATbio) in health workers, considering the isolated and combined analysis of the dimensions of two models, the demand-control model (DCM) and the effort-reward imbalance model (ERI).

**METHODS:**

Cross-sectional study in a representative sample of workers with higher, technical and secondary education, including health agents from primary and medium-complexity care units in five cities in Bahia. Random sampling was selected, stratified by geographic area, level of service complexity and occupation. The outcome variable was ATbio; The main exposure was occupational stressors, assessed by the DCM and ERI. Incidences and relative risks were estimated as a function of the acute, short-term nature of the outcome of interest. Associations between ATbio and isolated and combined DCM and ERI dimensions were tested.

**RESULTS:**

A total of 3,084 workers participated in the study. The global incidence of ATbio was 3.4% and was associated with high psychological demand, high effort and high commitment to work, adjusted for sex, age, education and work shift. High-strain work and a situation of imbalance between efforts and rewards were associated with ATbio. With the combination of the models, an increase in the measure of association with the outcome was observed. Significant associations of greater magnitude were observed in the complete combined models. ATbio’s risk was 5.23 times higher among those exposed in both complete models compared to the absence of exposure in both models.

**CONCLUSIONS:**

Occupational stressors were associated with ATbio. Advantages in using the combined models were observed. The approach of different psychosocial dimensions has expanded the ability to identify exposed groups, offering a solid basis for interventions for ATbio’s prevention in health.

## INTRODUCTION

A typical work accident is defined as an injury that occurs during work hours in the service of a company, causing bodily injury or functional disturbance that can cause loss or reduction (permanent or temporary) in the ability to work or even lead to death^1^. This type of injury is one of the most important events in the occupational health area, both in number of cases and in the severity of occurrences, impacting the morbidity and mortality rates of the working population.

For health workers, accidents due to exposure to biological material (ATbio) are more frequent. Contact with blood and body fluids exposes these workers to the risk of contamination by various pathogens, including hepatitis B and C and human immunodeficiency viruses^2,3^. ATbio is associated with the worker’s age, work sector, infection prevention training, use of protective equipment (such as gloves and glasses), presence of a safety committee, signalization, vaccination for infectious diseases and needle recapping after its use^4^.

In addition to biosafety-related factors, a high level of occupational stress is the risk factor for work accidents^5^. Characteristics related to the activity of health workers (tension caused by task speed, time pressure, conflicts in social relationships, repetitive and fragmented tasks, high psychological demands and complex cognitive skills) expose these professionals to ATbio. However, despite the growing recognition that work psychosocial aspects (especially occupational stressors) are associated with a higher risk of ATbio, this relation is rarely privileged in the analysis of risk situations. In other words, although they are always included in the list of risk factors, the number of studies that explored or deepened these aspects is still limited, especially in Brazil^10^.

Over the years, different models, applied to epidemiological studies, have been used to measure occupational stressors^11^. One can mention the demand-control model – DCM^12^, which highlights labor demands and the degree of autonomy involved, and the effort-reward imbalance model – ERI^13^, which considers situations of balance and reciprocity in work relationships, focusing on aspects related to efforts and rewards.

The DCM highlights two aspects capable of triggering occupational stress: psychological demand and control over work. Demands are related to the quantity, excess and pace of work to be performed, insufficient time to perform tasks and work done under pressure. Control refers to the worker’s mastery of the task and the possibility of defining the organization of the work itself^14^. The combination of these two dimensions distinguishes four work situations, structured around the combination of high/low psychological demand and high/low level of control. The experience of “high demand” (which combines high demand and low control) represents a greater risk to the worker’s health.

The dimension “social support from colleagues and management in the work environment” was incorporated to the two-dimensional model (demand and control), since the two initial dimensions were insufficient to elucidate the complex relationship between the psychosocial aspects of work and illness, thus proposing a three-dimensional model (demand, control and social support)^15^. The hypothesis for this incorporation is that social support at work exerts a moderating effect on the occupational strain experienced in stressful situations, which can reduce, eliminate or increase potential negative impacts on health.

In the ERI model, it is postulated that the non-reciprocity between efforts at work and the low rewards received, that is, the imbalance in this relationship, can generate stressful situations, predisposing the individual to mental suffering. In this model, the effort refers to the demands perceived by the worker in performing the tasks, and the reward, in turn, alludes to gains arising from this effort (financial gain, self-esteem and status). The hypothesis is that work characterized by high effort and low reward is harmful to health, causing illness and harm. To this two-dimensional model, a third dimension was also incorporated, the excessive commitment to work (CET), which corresponds to the situation of “over dedication”, an intrinsic characteristic of the worker, increasing the negative effects on health.

Therefore, each model addresses the impact of occupational stressors on workers’ health in a different way, emphasizing specific characteristics. Thus, individually, such models may have limits to explain the complexity of the relationship between stressors and health problems. Considering this, some studies have shown increased predictive power when the dimensions of these models are combined in the analysis of different outcomes^16^.

In Brazil, as already mentioned, only a few researches that assess the effects of occupational stressors on ATbio^10^exists, and no studies that used combined models to measure this association were found. Considering the potential effects of ATbio on workers’ health, the importance of broadening the discussion on the impact of occupational stressors on the occurrence of these accidents is highlighted. This discussion can support more comprehensive prevention strategies and with greater potential to protect workers’ health.

The aim of this study was to evaluate the association between occupational stressors and ATbio in health workers, considering the isolated and combined analysis of the dimensions of two models, the DCM and the ERI. Thus, in addition to evaluating the relation of occupational stressors with ATbio, it will be possible to verify whether the combined analysis of the models’ dimensions contributes to a more comprehensive ATbio risk assessment, with greater capacity to identify harmful psychosocial situations that increase the risk of accidents.

## METHODS

The analyzed data come from a cross-sectional study that is part of the multicentric research “Condições de trabalho, condições de emprego e saúde dos trabalhadores da saúde na Bahia”, carried out in the municipalities of Feira de Santana, Itabuna, Jequié, Santo Antônio de Jesus and Centro Histórico de Salvador, developed by the Epidemiology Center of the Universidade Estadual de Feira de Santana.

The population consisted of health workers in primary and medium-complexity care from the cities mentioned, regardless of the type of employment relationship. The study included higher education workers (doctors, nurses, physiotherapists, dentists, psychologists, social workers), technical education workers (nursing technicians, dental assistants) and secondary school education workers (general services, administrative and surveillance personnel) and health agents (community health and endemic diseases agents). Workers were selected by random sampling, stratified by geographic area (coverage area of the Extended Family Health Centers), level of service complexity and professional category.

To calculate the sample size, the total number of primary and medium-complexity care workers (n = 6,191) were considered, assuming the estimated incidence of the event of interest, ATbio, of 11.9%^21^, accuracy of 3%, with a 95% confidence interval. From these parameters, a sample size of 418 individuals was established, with an increase of 20% (n = 84), already considering possible losses. The final sample, therefore, was estimated at 502 workers.

Data were collected in 2012 by interviews at the participants’ workplace, conducted by trained interviewers. Workers on leave or on vacation or who had less than six months of work time were excluded. The strategy of three visits was adopted to carry out the interview. In case of failure after these attempts, the worker was replaced by a draw, respecting the characteristics of geographic area, complexity of health services, professional category and sex.

The questionnaire used, based on the literature review, was previously tested in a pilot study. The instrument included sociodemographic (sex, age, children, marital status, educational level, skin color and income), occupational (psychosocial aspects, shift and working hours) and lifestyle (physical activities, leisure, smoking and consumption of alcoholic beverages) characteristics.

The study outcome variable, “work accidents with exposure to biological material”, was evaluated by the question: “In the last 12 months, have you suffered any work accident that put you in direct contact with blood, sputum or other body fluids from a patient?”. The answer was categorized as “yes” and “no”. The 12-month time cut was established to reduce recall bias, considering that the research was self-reported.

The psychosocial aspects of work (occupational stressors) comprised the main exposure variable, measured using the DCM/AST (social support at work) and ERI/CET (overcommitment to work) models, using the Job Content Questionnaire (JCQ) and the ERI (effort-reward imbalance), respectively. The JCQ was translated and validated for use in occupational groups in Brazil, showing a good overall performance^22^. The ERI showed adequacy of psychometric performance in a study conducted with nursing professionals in Brazil^23^.

In the DCM, the JCQ full version was used, including 5 items of psychological demand, 9 of control over work and 6 of social support at work (AST). The scores (sum of the items that make up each dimension), as well as the ratio between demand and control (D/C), were categorized by the median as “high” (≥ median) and “low” (< median). Thus, it was possible to establish the four work experiences provided for in the DCM: “high demand” (high demand and low control), “active work” (high demand and high control), “passive work” (low demand and low control) and “low demand” (low demand and high control).

The short version of the ERI was used, including the effort (3 items), reward (7 items) and CET (6 items) scales. The scores of the three dimensions were categorized by the median as “high” (≥ median) and “low” (< median). The effort-reward imbalance indicator was obtained from the formula (e/r)*c, where “*e*” is the sum of the effort items, “*r*” corresponds to the sum of the reward items, and “*c*” is a correction factor, considering the number of items in the numerator compared to the denominator. Thus, in the effort-reward ratio, values > 1 were considered as a stressful situation, since the effort was greater than the reward, indicating an imbalance between these dimensions.

The dimensions of the models, including the number of items in the DCM and ERI, variation in scores and respective medians, are presented in the Box. As described in the chart, the DCM without AST was called “partial DCM” and, with AST, “complete DCM”. The ERI model without CET corresponded to the “partial ERI” model and, with the CET, the “complete ERI” model. To analyze the effect of the combined partial model, the partial DCM (demand and control, without AST) and the partial ERI (effort and reward, without CET) were used. The complete DCM (which included the AST) and the complete ERI (with CET) were used to analyze the combined complete model (Box).

**Box:** Dimensions of the demand-control model (DCM) and effort-reward imbalance model (ERI), including number of items, score variation, median and composition of each partial and complete analysis model.

Dimensions (Abbreviation)	No. of items	Variation	Median	Partial model	Complete model
Demand-control model				DC	DC + AST
Psychological demand (D)	5	12–48	28.0	Yes	Yes
Control over work (C)	9	24–96	64.0	Yes	Yes
Social support at work (AST)	6	6–24	18.0	No	Yes
Effort-reward imbalance model				ERI	ERI + CET
Effort (E)	3	3–12	10.0	Yes	Yes
Reward (R)	7	7–28	16.0	Yes	Yes
Excessive commitment to work (CET)	6	6–24	14.0	No	Yes

^a^ Combined partial model: [DC] + [ERI].

^b^ Combined complete model: [DC + AST] + [ERI + CET].

Data analysis included the calculation of incidence and relative risks (RR) and their respective 95% confidence intervals. Although cross-sectional studies are indicated to analyze prevalent cases, it is possible, in some cases, based on information reported in the past, to estimate the incidence^24^. In this study, cases of work accidents were considered as incidents, since they are events circumscribed in time, of a sudden, acute and short-term nature^25^.

In the descriptive analysis of the data, sociodemographic characteristics and lifestyle habits were considered to outline the sample’s profile, as well as to estimate the outcome’s incidence. Then, by bivariate analysis, the following were evaluated: gross association between each dimension of the models with the outcome; association between each partial model (DCM and ERI) with the outcome; association between each complete model (DCM/AST and ERI/CET) with the outcome; and association between the combination of partial models with the outcome, according to procedures performed by Griep et al.^17^. The study also evaluated the association between the combination of complete models with the outcome.

For the combined models, workers were categorized into four groups, considering exposure in one or another dimension. The group not exposed in any of the models was considered as a reference category. To build the categories, the scores for each exposure were dichotomized according to tertiles (1^st^ and 2^nd^: absence of exposure; and 3^rd^: presence of exposure).

To test for confounding, a stratified analysis was performed, which included sociodemographic and occupational covariates. Gross and adjusted measures of association were compared. Variables that presented differences in these measures with a variation above 20% were considered potential confounders. Findings in the literature were also weighted to select these variables. Thus, for the final models, the variables “sex”, “age”, “educational level” and “work shift” were added in the modeling to adjust for confounding.

In the multivariate analysis, the covariates considered confounding were included. At this stage, Poisson regression with robust variance was used to estimate the adjusted measures of association (_adjusted_RR) and respective 95% confidence intervals. Although Poisson regression was originally aimed at quantitative outcomes (counts), it can also be used to model data with binary outcomes, with appropriate methods (e.g., the robust variance used in this analysis), providing valid estimates of risk and confidence levels^26^.

Data entry and database cleaning were performed using the Statistical Package for Social Science (SPSS) software, version 17.0, for Windows. To analyze the data, the Stata software, version 12.0, was used.

The project, approved by the Research Ethics Committee of the Universidade Estadual de Feira de Santana under Protocol No. 081/2009 and CAAE No. 50801715.3.0000.0053, meets the specifications of Resolutions No. 466/2012 and No. 510/2016 of the Brazilian National Health Council. All workers who agreed to participate in the study signed an informed consent form.

## RESULTS

At the end of the research, 3,084 health workers were interviewed. Most were female (78.2%), aged up to 39 years old (55%), with children (68.8%), partner (57.3%), intermediate or technical education (53%), black or brown skin color (80.6%) and monthly income of up to three minimum wages (78.2%). Regarding lifestyle habits, most reported performing leisure (83.6%) and physical (52.5%) activities. Smoking was reported by 17.6% of workers, and alcohol consumption by 39.7% (Table 1).

**Table 1:** Distribution of health workers according to sociodemographic characteristics and lifestyle habits, Bahia, Brazil, 2012.

Characteristics^a^	n	%
Sociodemographic		
Sex (n = 3,077)		
Female	2,405	78.2
Male	672	21.8
Age group (n = 3,061)		
Up to 39 years old	1,683	55.0
More than 39 years old	1,378	45.0
Children (n = 3,065)		
Yes	2,108	68.8
No	957	31.2
Marital status (n = 3,074)		
Without a partner	1,314	42.7
With a partner	1,760	57.3
Educational level (n = 3,042)		
Elementary education	122	4.0
Secundary or technical education	1,611	53.0
Higher education (complete or not)	1,309	43.0
Skin color (n = 3,032)		
Black	2,444	80.6
Non-black	588	19.4
Monthly income (n = 2,560)^b^		
Up to 3 minimum wages	2,003	78.2
More than 3 minimum wages	557	21.8

Lifestyle habits		
Leisure activity (n = 3,058)		
No	503	16.4
Yes	2,555	83.6
Physical activity (n = 3,048)		
No	1,449	47.5
Yes	1,599	52.5
Smoking habit (n = 3,020)		
Yes	533	17.6
No	2,487	82.4
Alcoholic beverage consumption (n = 2,514)		
Yes	998	39.7
No	1,516	60.6

^a^ The n varied depending on the loss of information on the analyzed variables.

^b^ Minimum wage in force at the time: R$ 622.00.

The global incidence of ATbio in the researched group was 3.4%. Statistically significant associations were found between ATbio and high psychological demand, high effort and high commitment to work, even with the adjustment for sex, age, educational level and work shift (Table 2).

**Table 2:** Incidence (%), gross and adjusted relative risk of work accidents, according to dimensions of the demand-control and effort-reward imbalance models, in health workers, Bahia, Brazil, 2012.

Dimensions	Work accidents				

	I%	RR	95%CI	RRª	95%CI
DCM/AST^b^					
Psychological demand					
High	4.4	1.71	1.17–2.52	1.60	1.07–2.40
Low	2.6	1.00			
Control over work					
Low	3.6	1.08	0.74–1.59	1.08	0.73–1.60
High	3.4	1.00			
Social support at work					
Low	3.7	1.31	0.83–2.08	1.24	0.78–1.96
High	2.8	1.00			

ERI/CET^c^					
Effort					
High	4.6	2.16	1.43–3.26	2.18	1.41–3.36
Low	2.1	1.00			
Reward					
Low	4.0	1.45	0.99–2.14	1.36	0.92–2.02
High	2.8	1.00			
Excessive commitment					
High	4.5	1.88	1.27–2.78	1.92	1.28–2.88
Low	2.4	1.00			

^a^ Relative risk adjusted for sex, age, educational level and work shift.

^b^ Demand-control/social support at work model.

^c^ Effort-reward imbalance/excessive commitment to work model.

^d^ P-value < 0.05.

Partial models (DCM without AST and ERI without CET) were associated with the outcome, except in passive work. Working conditions with high strain and imbalance between efforts and rewards were associated with ATbio in the partial models. By incorporating the third dimension to the DCM and the ERI (complete models), there was an association with ATbio, with measures of greater magnitude, when compared to the partial models. ERI showed a stronger magnitude of association with ATbio compared to compared to DCM (Table 3).

**Table 3:** Incidence (%), gross and adjusted relative risk of work accidents, according to partial, complete and combined models of demand-control and effort-reward imbalance, in health workers, Bahia, Brazil, 2012.

Model	Work accidents				

	I%	RR	95%CI	RRª	95%CI
Partial models					
DCM^b^					
High demand	4.5	1.87	1.06–3.28	1.81	1.01–3.24
Passive work	3.1	1.29	0.72–2.32	1.51	0.83–2.75
Active work	4.5	1.87	1.06–3.29	2.06	1.14–3.71
Low demand	2.4	1.00			
ERI^c^					
Imbalance	5.3	2.24	1.52–3.30	2.10	1.42–3.11
Balance	2.4	1.00			

Complete models					
DCM/AST^d^					
DC and AST (exposure in both)	4.4	2.12	1.10–4.08	1.97	1.03–3.78
Exposure in DC	4.9	2.35	1.05–5.26	2.45	1.10–5.46
Exposure in AST	3.1	1.47	0.74–2.92	1.39	0.70–2.78
Not exposed	2.1	1.00			
ERI/CET^e^					
ERI and CET (exposure in both)	5.7	3.01	1.83–4.95	3.01	1.80–5.02
Exposure in ERI	4.3	2.27	1.17–4.38	2.31	1.18–4.50
Exposure in CET	3.4	1.76	1.01–3.08	1.98	1.13–3.46
Not exposed	1.9	1.00			

Combined Partial Model					
DCM and ERI					
DC and ERI (exposure in both)	6.8	3.08	1.88–5.06	2.92	1.78–4.79
Exposure in ERI	3.8	1.74	0.93–3.25	1.71	0.92–3.19
Exposure in DC	2.8	1.25	0.71–2.23	1.39	0.78–2.49
Not exposed	2.2	1.00			

Combined Complete Model					
DCM/AST and ERI/CET					
DC/AST and ERI/CET (exposure in both)	8.2	5.83	2.04–16.7	5.23	1.82–15.1
Exposure in DC/AST	2.0	1.42	0.42–4.80	1.79	0.59–5.45
Exposure in ERI/CET	1.3	0.92	0.11–8.13	0.96	0.13–6.80
Not exposed	1.4	1.00			

DCM: demand-control model; DC: demand-control; AST: social support at work; ERI: effort-reward imbalance model; CET: excessive commitment to work.

^a^ Relative risk adjusted for sex, age, educational level and work shift.

^b^ P-value < 0.05.

In the combination of the partial models, an association between the focused dimensions and the outcome was observed. The risk of ATbio was 2.92 times higher among those exposed in both models when compared to the absence of exposure in both models, even after the adjustment for confounding variables. The frequency of accidents was higher when analyzing the combined partial model compared to isolated partial models (Table 3).

When combining the complete models, a greater magnitude of association with the outcome was observed compared to the other models – isolated partial and complete and combined partial. The risk of ATbio was 5.23 times higher among those exposed in both complete models, compared to the absence of exposure in both models, even after the adjustment (Table 3).

## DISCUSSION

The incidence of occupational accidents with exposure to biological material was lower than that found in other studies^29^. When considering that the occurrence of the outcome was mentioned by the worker, one must consider the possibility of recall bias and that accidents judged as minor and irrelevant have not been considered. In a research carried out at a university hospital, considering an accident as low risk has already been identified as a cause of underreporting of biological accidents by nursing professionals^32^. Therefore, a probable underestimation must be considered in the analysis of the result found, due to a certain naturalization of the risk.

Exposure to high psychological demand was associated with work accidents, as observed in other investigations^6,7,33^. Performing tasks at a fast pace and in insufficient time can reduce attention, increasing exposure to occupational hazards and work accidents, especially with biological material in the case of health workers.

The “control over one’s own work” dimension, separately analyzed, was not associated with accidents. As the control over work in the studied group (health workers) is relatively high, due to the demands of work requiring the use of skills and relative decision-making authority, perhaps the variability was not wide enough to capture possible differences. The scores’ similarity of a measure between defined groups makes it difficult to observe significant differences. This has been reported in researches in the worker health area when certain job characteristics have little internal variability in the occupational group under analysis. In any case, when low control was associated with high psychological demand, exposure to stressful conditions and the effect on the occurrence of accidents increased. High strain situation (high demand and low control simultaneously) was also associated with work leave due to accidents in other studies^34^.

Health work is characterized by the complexity of tasks and responsibility for the other. The worker’s attention can be dispersed when the functions are performed at an intense pace, under time pressure, with task overload and frequent experience of unforeseen or conflicting situations that require a high level of knowledge and resources and with the performance of multiple tasks^37^. In these cases, stress affects the ability to concentrate, hindering self-care actions and increasing the risk of work accidents, especially when it comes to tasks that require greater skill and dexterity, such as handling sharps objects.

It is not rare that the worker, subject to precarious conditions (insufficient human and material resources), has to attend a large number of users in a short period of time, with different demands, which forces them to develop multiple and repetitive tasks, and not always with the necessary autonomy for proper decision-making. These stressful situations, a characteristic of highly demanding work, wear out the professional, favoring negligence in carrying out tasks and, consequently, increasing the occurrence of work accidents.

In the ERI model, the reward, which was not associated with the outcome of interest, refers to the gains provided by work (such as adequate salary, respect and support from colleagues, career promotions and job stability). However, in health work, the reward seems to be more associated with symbolic values: care for the other and the affection and bond established in relationships. Thus, the reward aspects assessed by the ERI may not have been adequate enough to detect these other forms of reward in health work.

The effort-reward imbalance was associated with work accidents. This disproportion increased the incidence of occupational injuries in a study with Korean workers^9^. The health worker deals with the pressure of time and the heavy workload, intensified by frequent interruptions and various difficulties faced during the journey. This can generate or aggravate concentration deficits in performing tasks, which increases the risk of accidents^37^. That is, not only the work environment can be inadequate, but also the psychosocial characteristics of the work which, producing situations of stress and weariness, constraints of time, resources and imbalance between efforts and rewards, can favor the occurrence of accidents. Intense work rhythms or excessive volume of tasks can also reduce the capacity for attention and concentration, increasing the worker’s vulnerability. Therefore, researches have reinforced the role of the psychosocial situation in work accidents^5^.

The incorporation of a third dimension (excessive commitment to work) in the ERI intensified the strength of the association with ATbio. Considered an intrinsic and subjective component of the model, “over dedication” involves high expectations of recognition. People in this situation tend to be excessively busy with their work, showing dependence on the feedback received and the approval of others^13,20^.

Due to its own characteristics (establishment of bonds, care and concern for other’s suffering), health work favors a high professional involvement, which can often generate excessive commitment to the activities performed. The experience of health work as a vocation, as a life mission, often leads professionals to overestimate the role of work in relation to other aspects of life^20,38^. Thus, intense demands on oneself are frequent, making it impossible to “disconnect” and experience other situations, which generates anxiety and reduces alertness, consequently increasing the occurrence of accidents. Furthermore, if the dedication is very high, the need for rewards also increases, and the frustration of this expectation can produce even more suffering. Areosa and Dwyer^39^ emphasize the importance of investigating the possible pressures present at the time of the accident, including anxiety, in order to better understand the mechanisms that connect emotions and negative experiences to work-related accidents and illnesses.

In the literature, social support has been shown to be important to mitigate the negative impacts of occupational stress on health workers, being a protective factor for general health^17,20^. In this study, the incorporation of social support at work into the DCM helped to improve the outcome prediction, as also observed in another study^17^. Health work is characterized by strong interpersonal relationships and collaboration among colleagues. Low social support associated with high-strain work worsens stressful situations.

In the partial and complete models, considering the specific measures of association, ERI showed the ability to identify more sensitive exposure situations than DCM. Karasek’s model^12^ is widely used in the literature to investigate the association between work stressors and the most varied outcomes. It was proposed in the 1970s to evaluate industrial work, with emphasis on aspects involved in carrying out the tasks, which brings difficulties for its application in health work. This work’s specific characteristics, based on complex human relationships, do not allow the mere transposition of the model based on the industrial production process^14^. Thus, Söderfeldt et al.^40^ point out the need to adapt dimensions of the DCM in order to enable its use in human services organizations. The ERI, by incorporating the reward at work, may have increased the model’s explanatory power in relation to working conditions in health compared to the MDC.

The combined use of the DCM and ERI models increased the predictive power of the relationship between stressors and work accidents. Other studies have also shown better performance in identifying unfavorable health situations with the combination of models, which seems to be a promising alternative to the limitations of using isolated models to encompass the complexity of the psychosocial work environment^18,20^.

These models address specific aspects of work (the DCM assesses elements related to the task, demands and degree of autonomy involved, while the ERI addresses issues of injustice, imbalance in work exchanges), but when used together, they allow a broader assessment of the characteristics and effects of different stressors present simultaneously. The data obtained reinforce the relevance of this combined use of models, which allows for a more comprehensive understanding of the work’s psychosocial aspects. Griep et al.^41^ observed that different combinations of stressors resulted in different predictions of absenteeism. Although the partial models have shown good performance in identifying risk situations for ATbio, the combination of dimensions addressed in the two models tested allowed the identification of situations of greater exposure, which deserve greater attention in the prevention of ATbio. Once these situations and groups that may be exposed are identified, the work and dimensions analyzed here can be reorganized, incorporating actions, for example, into biosafety programs.

Some limitations of the present study must be highlighted. Self-reported measures, as already mentioned, can be influenced by recall bias. Furthermore, since the investigation was conducted with workers who were in full exercise of their work activities, the effect of the healthy worker cannot be disregarded^42^. Thus, professionals on leave as a result of work accidents or other health problems of occupational origin may not have participated in the research.

Despite these potential limitations, the results obtained reinforce the relevance of psychosocial aspects of work in exposure to ATbio. There was also evidence of an increase in the explanatory potential for ATbio when partial and complete models are used in combination, which is an important finding of this study. The analysis of several dimensions of work expanded the understanding of the connections that generate situations of vulnerability to ATbio, with detailed analysis of those related to the work’s psychosocial structure.

It is concluded that, due to the different dimensions addressed by the models used (which complement each other and allow the identification of occupational exposures in more detail), their combined use can support more effective interventions in health work environments and processes, aiming at the prevention of ATbio and other health problems not addressed in this study. In other words, the results show the need for ATbio prevention programs that are not limited only to the control of biosafety aspects, but also incorporate psychosocial aspects of work in the broadest possible way.
