# Supplementary material for: Occupational stressors and work accidents among health workers
Source: Rev Saude Publica. 2021 Dec 2;55:98. doi: 10.11606/s1518-8787.2021055002938 (PMC8664054; doi:10.11606/s1518-8787.2021055002938)
Supplement: Translation

# Translation: Estressores ocupacionais e acidentes de trabalho entre trabalhadores da saúde

**Authors:** Mariana Rabelo Gomes, Tânia Maria de Araújo, Jorgana Fernanda de Souza Soares, Camila Carvalho de Sousa, Iracema Lua

**Affiliations:** I Brasil Universidade Federal da Bahia. Instituto de Saúde Coletiva. Programa de Pós-Graduação em Saúde Coletiva. Salvador, BA, Brasil; II Brasil Universidade Estadual de Feira de Santana. Departamento de Saúde. Feira de Santana, BA, Brasil; III Brasil Universidade Federal da Bahia. Faculdade de Medicina da Bahia. Programa de Pós-Graduação em Saúde, Ambiente e Trabalho. Salvador, BA, Brasil; IV Brasil Universidade Estadual de Feira de Santana. Programa de Pós-Graduação em Saúde Coletiva. Feira de Santana, BA, Brasil

**Keywords:** Pessoal de Saúde, psicologia, Acidentes de Trabalho, prevenção & controle, Exposição Ocupacional, Contenção de Riscos Biológicos, Capacitação em Serviço

## Abstract

**OBJETIVO:**

Testar associação entre estressores ocupacionais e acidentes de trabalho por exposição a material biológico (ATbio) em trabalhadores da saúde, considerando a análise isolada e combinada das dimensões de dois modelos, o modelo demanda-controle (MDC) e o modelo de desequilíbrio esforço-recompensa (ERI).

**MÉTODOS:**

Estudo de corte transversal em amostra representativa de trabalhadores de nível superior, técnico, médio, incluindo agentes de saúde de unidades da atenção básica e média complexidade em cinco cidades da Bahia. Selecionou-se amostragem aleatória, estratificada por área geográfica, nível de complexidade do serviço e ocupação. A variável desfecho foi ATbio; a exposição principal foram os estressores ocupacionais, avaliados pelo MDC e ERI. As incidências e os riscos relativos foram estimados em função do caráter agudo, de curta duração, do desfecho de interesse. Testaram-se associações entre ATbio e as dimensões do MDC e ERI isolados e combinados.

**RESULTADOS:**

Participaram do estudo 3.084 trabalhadores. A incidência global de ATbio foi de 3,4% e mostrou-se associada a alta demanda psicológica, alto esforço e alto comprometimento com o trabalho, com ajuste por sexo, idade, escolaridade e turno de trabalho. Trabalho de alta exigência e situação de desequilíbrio entre esforços e recompensas estavam associados aos ATbio. Com a combinação dos modelos, observou-se incremento da medida de associação com o desfecho. As associações significantes e de maior magnitude foram observadas nos modelos combinados completos. O risco de ATbio foi 5,23 vezes maior entre os expostos em ambos os modelos completos em comparação com a ausência de exposição nos dois modelos.

**CONCLUSÕES:**

Estressores ocupacionais mostraram-se associados aos ATbio. Observaram-se vantagens no uso dos modelos combinados. A abordagem de diferentes dimensões psicossociais ampliou a capacidade de identificação de grupos expostos, oferecendo base sólida para intervenções de prevenção dos ATbio em saúde.

## INTRODUÇÃO

Acidente de trabalho típico é definido como um agravo que ocorre durante o exercício do trabalho a serviço de uma empresa, provocando lesão corporal ou perturbação funcional que pode causar perda ou redução (permanente ou temporária) da capacidade para o trabalho ou até levar à morte^1^. Esse tipo de agravo é um dos mais importantes eventos na área da saúde do trabalhador, tanto em número de casos quanto na gravidade das ocorrências, impactando as taxas de morbimortalidade da população trabalhadora.

Para os trabalhadores da saúde, os acidentes por exposição a material biológico (ATbio) são mais frequentes. O contato com sangue e fluidos orgânicos expõe esses trabalhadores a risco de contaminação por diversos patógenos, dentre eles os vírus das hepatites B e C e da imunodeficiência humana^2,3^. Associam-se aos ATbio a idade do trabalhador, setor de trabalho, treinamento para prevenção de infecções, uso de equipamentos de proteção (como luvas e óculos), presença de comitê de segurança, sinalização, vacinação para as doenças infecciosas e recapagem de agulhas após o uso^4^.

Além de fatores relacionados à biossegurança, alto nível de estresse ocupacional é fator de risco para acidentes de trabalho^5^. Características relacionadas à atividade dos trabalhadores da saúde (tensão causada pela velocidade das tarefas, pressão do tempo, conflitos nas relações sociais, tarefas repetitivas e fragmentadas, alta exigência psicológica e habilidades cognitivas complexas) expõem esses profissionais a ATbio. No entanto, apesar do crescente reconhecimento de que os aspectos psicossociais do trabalho (especialmente os estressores ocupacionais) associam-se a maior risco de ATbio, raramente essa relação é privilegiada na análise de situações de risco. Ou seja, embora sejam sempre incluídos na lista de fatores de risco, o número de estudos que exploraram ou aprofundam esses aspectos é ainda restrito, especialmente no Brasil^10^.

Ao longo dos anos, diferentes modelos, aplicados aos estudos epidemiológicos, têm sido utilizados para mensurar estressores ocupacionais^11^. Pode-se citar o modelo demanda-controle (*demand-control model* – MDC)^12^, que destaca as demandas laborais e o grau de autonomia envolvidos, e o modelo de desequilíbrio esforço-recompensa (*effort-reward imbalance model* – ERI)^13^, que considera situações de equilíbrio e reciprocidade nas relações no trabalho, focalizando aspectos relacionados aos esforços e às recompensas.

O MDC destaca dois aspectos capazes de desencadear estresse ocupacional: a demanda psicológica e o controle sobre o trabalho. As demandas estão relacionadas à quantidade, ao excesso e ao ritmo de trabalho a ser realizado, ao tempo insuficiente para executar as tarefas e ao trabalho feito sob pressão. O controle refere-se ao domínio do trabalhador sobre a tarefa e a possibilidade de definir a organização do próprio trabalho^14^. A combinação dess as duas dimensões distingue quatro situações no trabalho, estruturadas com base na combinação de alta/baixa demanda psicológica e alto/baixo nível de controle. A experiência de “alta exigência” (que conjuga alta demanda e baixo controle) representa maior risco para a saúde do trabalhador.

Ao modelo bidimensional (demanda e controle), foi incorporada a dimensão “apoio social de colegas e chefia no ambiente de trabalho”, já que as duas dimensões iniciais eram insuficientes para elucidar a complexa relação entre os aspectos psicossociais do trabalho e o adoecimento, propondo-se, assim, um modelo tridimensional (demanda, controle e apoio social)^15^. A hipótese para essa incorporação é que o apoio social no trabalho exerce efeito moderador sobre o desgaste ocupacional vivenciado em situações estressoras, podendo reduzir, eliminar ou ampliar os potenciais impactos negativos sobre a saúde.

No modelo ERI postula-se que a não reciprocidade entre os esforços no trabalho e as baixas recompensas recebidas, ou seja, o desequilíbrio nessa relação, pode gerar situações estressantes, predispondo o indivíduo ao sofrimento mental. Neste modelo, o esforço se refere a demandas percebidas pelo trabalhador na execução das tarefas, e a recompensa, por sua vez, alude a ganhos advindos desse esforço (ganho financeiro, autoestima e status). A hipótese é que o trabalho caracterizado por elevado esforço e baixa recompensa é prejudicial à saúde, produzindo adoecimento e agravos. A esse modelo bidimensional, também foi incorporada uma terceira dimensão, o comprometimento excessivo com o trabalho (CET), que corresponde à situação de “super dedicação”, característica intrínseca do trabalhador, potencializando os efeitos negativos sobre a saúde.

Cada modelo, portanto, aborda o impacto dos estressores ocupacionais na saúde dos trabalhadores de modo distinto, enfatizando características específicas. Assim, individualmente, tais modelos podem ter limites para explicar a complexidade da relação entre estressores e agravos à saúde. Considerando isso, alguns estudos têm demonstrado aumento do poder preditivo quando as dimensões desses modelos são combinadas na análise de diversos desfechos^16^.

No Brasil, como já mencionado, ainda são escassas as pesquisas que avaliam os efeitos dos estressores ocupacionais sobre os ATbio^10^, e não foram localizados estudos que utilizaram modelos combinados para mensurar essa associação. Considerando os potenciais efeitos dos ATbio na saúde dos trabalhadores, destaca-se a importância de ampliar a discussão sobre o impacto dos estressores ocupacionais na ocorrência desses acidentes. Essa discussão pode subsidiar estratégias de prevenção mais abrangentes e com maior potencial de proteger a saúde dos trabalhadores.

O objetivo deste estudo foi avaliar a associação entre estressores ocupacionais e ATbio em trabalhadores da saúde, considerando a análise isolada e combinada das dimensões de dois modelos, o MDC e o ERI. Desse modo, além de avaliar a relação dos estressores ocupacionais com ATbio, será possível verificar se a análise combinada das dimensões dos modelos contribui para uma avaliação de riscos de ATbio mais abrangente, com maior capacidade de identificar situações psicossociais nocivas que elevam o risco de acidentes.

## MÉTODOS

Os dados analisados são oriundos de estudo de corte transversal. Trata-se de um recorte da pesquisa multicêntrica “Condições de trabalho, condições de emprego e saúde dos trabalhadores da saúde na Bahia”, realizada nos municípios de Feira de Santana, Itabuna, Jequié, Santo Antônio de Jesus e Centro Histórico de Salvador, desenvolvida pelo Núcleo de Epidemiologia da Universidade Estadual de Feira de Santana.

A população foi composta por trabalhadores da saúde da atenção básica e de média complexidade dos municípios citados, independentemente do tipo de vínculo empregatício. O estudo incluiu trabalhadores de nível superior (médicos, enfermeiros, fisioterapeutas, dentistas, psicólogos, assistentes sociais), de nível técnico (técnicos de enfermagem, auxiliares de odontologia) e médio (serviços gerais, pessoal administrativo e de vigilância) e agentes de saúde (agentes comunitários de saúde e de endemias). Os trabalhadores foram selecionados por amostragem aleatória, estratificada por área geográfica (área de cobertura dos Núcleos Ampliados de Saúde da Família), nível de complexidade do serviço e categoria profissional.

Para calcular o tamanho amostral, considerou-se o total de trabalhadores da atenção básica e da média complexidade (n = 6.191), assumindo-se a incidência estimada do evento de interesse, ATbio, de 11,9%^21^, precisão de 3%, com intervalo de confiança de 95%. A partir desses parâmetros, foi estabelecido tamanho amostral de 418 indivíduos com acréscimo de 20% (n = 84), já considerando possíveis perdas. A amostra final, portanto, foi estimada em 502 trabalhadores.

Os dados foram coletados em 2012, por meio de entrevistas no local de trabalho dos participantes, conduzidas por entrevistadores treinados. Excluíram-se os trabalhadores afastados por licença ou férias ou que contavam com menos de seis meses de atuação. Adotou-se a estratégia de três visitas para realização da entrevista. No caso de insucesso após essas tentativas, houve substituição, por sorteio, do trabalhador, respeitando-se as características de área geográfica, complexidade do serviços de saúde, categoria profissional e sexo.

O questionário empregado, construído com base na revisão de literatura, foi previamente testado em estudo-piloto. O instrumento incluiu características sociodemográficas (sexo, idade, filhos, situação conjugal, escolaridade, cor da pele e renda), ocupacionais (aspectos psicossociais, turno e jornada de trabalho) e de hábitos de vida (atividades físicas, lazer, tabagismo e consumo de bebidas alcoólicas).

A variável-desfecho do estudo, “acidentes de trabalho com exposição a material biológico”, foi avaliada pela pergunta: “Nos últimos 12 meses, você sofreu algum acidente de trabalho que o colocou em contato direto com sangue, escarro ou outros líquidos corporais do paciente?”. A resposta foi categorizada em “sim” e “não”. O corte temporal de 12 meses foi estabelecido para reduzir o viés de memória, considerando que a pesquisa se baseou em relato autorreferido.

Os aspectos psicossociais do trabalho (estressores ocupacionais) compuseram a variável de exposição principal, mensurada a partir dos modelos MDC/AST (apoio social no trabalho) e ERI/CET (comprometimento excessivo com o trabalho), utilizando os instrumentos *Job Content Questionnaire* (JCQ – questionário sobre conteúdo do trabalho) e o ERI (desequilíbrio esforço-recompensa), respectivamente. O JCQ foi traduzido e validado para uso em grupos ocupacionais no Brasil, apresentando bom desempenho geral^22^. O ERI apresentou adequação do desempenho psicométrico em estudo conduzido com profissionais de enfermagem no Brasil^23^.

No MDC, a versão completa do JCQ foi utilizada, incluindo 5 itens de demanda psicológica, 9 de controle sobre o trabalho e 6 de AST. Os escores (somatório dos itens que compõem cada dimensão), assim como a razão entre demanda e controle (D/C), foram categorizados pela mediana em “alto” (≥ mediana) e “baixo” (< mediana). Assim, foi possível estabelecer as quatro experiências do trabalho previstas no MDC: “alta exigência” (alta demanda e baixo controle), “trabalho ativo” (alta demanda e alto controle), “trabalho passivo” (baixa demanda e baixo controle) e “baixa exigência” (baixa demanda e alto controle).

Utilizou-se a versão reduzida do ERI, incluindo as escalas de esforço (3 itens), recompensa (7 itens) e CET (6 itens). Os escores das três dimensões foram categorizados pela mediana em “alto” (≥ mediana) e “baixo” (< mediana). Obteve-se o indicador de desequilíbrio esforço-recompensa a partir da fórmula (e/r)*c, onde “*e”* é a soma dos itens de esforço, “*r”* corresponde à soma dos itens de recompensa, e “*c”* é um fator de correção, considerando a quantidade de itens do numerador comparado ao denominador. Assim, na razão esforço-recompensa, valores > 1 foram considerados como situação estressora, uma vez que o esforço foi maior que a recompensa, indicando desequilíbrio entre essas dimensões.

As dimensões dos modelos, incluindo número de itens do MDC e ERI, variação dos escores e respectivas medianas, são apresentadas no Quadro. Como descrito no quadro, o MDC sem AST foi denominado “MDC parcial” e, com o AST, “MDC completo”. O modelo ERI sem CET correspondeu ao modelo “ERI parcial” e, com o CET, modelo “ERI completo”. Para analisar o efeito do modelo parcial combinado, foram utilizados o MDC parcial (demanda e controle, sem AST) e o ERI parcial (esforço e recompensa, sem CET). O MDC completo (que incluiu o AST) e o ERI completo (com CET) foram utilizados para analisar o modelo completo combinado (Quadro).

**Quadro:** Dimensões do modelo demanda-controle (MDC) e modelo desequilíbrio esforço-recompensa (*effort-reward imbalance* – ERI), incluindo número de itens, variação dos escores, mediana e composição de cada modelo de análise parcial e completo.

Dimensões (Sigla)	N^o^ de itens	Variação	Mediana	Modelo parcial^a^	Modelo completo^b^
Modelo demanda-controle				DC	DC + AST
Demanda psicológica (D)	5	12–48	28,0	Sim	Sim
Controle sobre o trabalho (C)	9	24–96	64,0	Sim	Sim
Apoio social no trabalho (AST)	6	6–24	18,0	Não	Sim
Modelo desequilíbrio esforço-recompensa				ERI	ERI + CET
Esforço (E)	3	3–12	10,0	Sim	Sim
Recompensa (R)	7	7–28	16,0	Sim	Sim
Comprometimento excessivo com o trabalho (CET)	6	6–24	14,0	Não	Sim

^a^ Modelo parcial combinado: [DC] + [ERI].

^b^ Modelo completo combinado: [DC + AST] + [ERI + CET].

A análise dos dados incluiu o cálculo da incidência e dos riscos relativos (RR) e seus respectivos intervalos de confiança de 95%. Embora os estudos transversais sejam indicados para analisar casos prevalentes, é possível, em alguns casos, com base em informações referidas no passado, estimar a incidência^24^. Neste estudo, consideraram-se os casos de acidentes de trabalho como incidentes, uma vez que são eventos circunscritos no tempo, de natureza súbita, aguda e de curta duração^25^.

Na análise descritiva dos dados, foram consideradas as características sociodemográficas e de hábitos de vida para delinear o perfil da amostra, bem como estimar a incidência do desfecho. Em seguida, por meio de análise bivariada, foram avaliadas: associação bruta entre cada dimensão dos modelos com o desfecho; associação entre cada modelo parcial (MDC e ERI) com o desfecho; associação entre cada modelo completo (MDC/AST e ERI/CET) com o desfecho; e associação entre a combinação dos modelos parciais com o desfecho, segundo procedimentos realizados por Griep et al.^17^. O estudo avaliou ainda a associação entre a combinação dos modelos completos com o desfecho.

Para os modelos combinados, os trabalhadores foram categorizados em quatro grupos, considerando a exposição em uma ou outra dimensão. O grupo não exposto em nenhum dos modelos foi considerado como categoria de referência. Para construir as categorias, os escores de cada exposição foram dicotomizados segundo os tercis (1º e 2º: ausência de exposição; e 3º: presença de exposição).

Para testar confundimento, realizou-se a análise estratificada, que incluiu as covariáveis sociodemográficas e ocupacionais. Compararam-se as medidas de associação bruta e ajustada. Variáveis que apresentaram diferenças dessas medidas com variação acima de 20% foram consideradas potenciais confundidoras. Os achados na literatura também foram ponderados para selecionar essas variáveis. Desta forma, para os modelos finais, as variáveis “sexo”, “idade”, “escolaridade” e “turno de trabalho” foram acrescentadas na modelagem para ajuste de confundimento.

Na análise multivariada, foram incluídas as covariáveis consideradas confundidoras. Nessa etapa, utilizou-se a regressão de Poisson com variância robusta para estimar as medidas de associação ajustadas (RR_ajustado_) e respectivos intervalos de confiança de 95%. Embora a regressão de Poisson seja originalmente destinada a desfechos quantitativos (contagens), também pode ser usada para modelar dados com resultados binários, com métodos apropriados (por exemplo, a variância robusta utilizada nesta análise), fornecendo estimativas válidas de risco e níveis de confiança^26^.

A entrada dos dados e a limpeza do banco foram realizadas no *software Statistical Package for the Social Science* (SPSS), versão 17.0, para Windows. Para analisar os dados, utilizou-se o *software* Stata, versão 12.0.

O projeto, aprovado pelo Comitê de Ética em Pesquisa da Universidade Estadual de Feira de Santana sob o Protocolo nº 081/2009 e CAAE nº 50801715.3.0000.0053, atende às especificações das Resoluções nº 466/2012 e nº 510/2016 do Conselho Nacional de Saúde. Todos os trabalhadores que aceitaram participar do estudo assinaram termo de consentimento livre e esclarecido.

## RESULTADOS

Foram entrevistados, ao final da pesquisa, 3.084 trabalhadores da saúde. A maioria era do sexo feminino (78,2%), com idade até 39 anos (55%), com filhos (68,8%), companheiro (57,3%), ensino médio ou técnico (53%), cor da pele preta ou parda (80,6%) e renda mensal de até três salários-mínimos (78,2%). Em relação aos hábitos de vida, a maioria referiu realizar atividades de lazer (83,6%) e físicas (52,5%). O hábito de fumar foi referido por 17,6% dos trabalhadores, e o consumo de bebidas alcoólicas por 39,7% (Tabela 1).

**Tabela 1:** Distribuição dos trabalhadores da saúde segundo características sociodemográficas e hábitos de vida, Bahia, Brasil, 2012.

Características^a^	n	%
Sociodemográficas		
Sexo (n = 3.077)		
Feminino	2.405	78,2
Masculino	672	21,8
Faixa etária (n = 3.061)		
Até 39 anos	1.683	55,0
Mais de 39 anos	1.378	45,0
Filhos (n = 3.065)		
Sim	2.108	68,8
Não	957	31,2
Situação conjugal (n = 3.074)		
Sem companheiro	1.314	42,7
Com companheiro	1.760	57,3
Escolaridade (n = 3.042)		
Fundamental	122	4,0
Médio ou técnico	1.611	53,0
Superior (completo ou não)	1.309	43,0
Cor da pele (n = 3.032)		
Negros	2.444	80,6
Não negros	588	19,4
Renda mensal (n = 2.560)^b^		
Até 3 salários-mínimos	2.003	78,2
Mais de 3 salários-mínimos	557	21,8

Hábitos de vida		
Atividade de lazer (n = 3.058)		
Não	503	16,4
Sim	2.555	83,6
Atividade física (n = 3.048)		
Não	1.449	47,5
Sim	1.599	52,5
Hábito de fumar (n = 3.020)		
Sim	533	17,6
Não	2.487	82,4
Bebidas alcoólicas (n = 2.514)		
Sim	998	39,7
Não	1.516	60,6

^a^ Os n variaram em função da perda de informação das variáveis analisadas.

^b^ Salário-mínimo vigente à época: R$ 622,00.

A incidência global de ATbio no grupo pesquisado foi de 3,4%. Foram encontradas associações estatisticamente significantes entre ATbio e alta demanda psicológica, alto esforço e alto comprometimento com o trabalho, mesmo com ajuste por sexo, idade, escolaridade e turno de trabalho (Tabela 2).

**Tabela 2:** Incidência (%), risco relativo bruto e ajustado de acidentes de trabalho, segundo dimensões dos modelos demanda-controle e desequilíbrio esforço-recompensa, em trabalhadores da saúde, Bahia, Brasil, 2012.

Dimensões	Acidentes de trabalho				

	I%	RR	IC95%	RRª	IC95%
MDC/AST^b^					
Demanda psicológica					
Alta	4,4	1,71	1,17–2,52	1,60	1,07–2,40
Baixa	2,6	1,00			
Controle sobre o trabalho					
Baixo	3,6	1,08	0,74–1,59	1,08	0,73–1,60
Alto	3,4	1,00			
Apoio social no trabalho					
Baixo	3,7	1,31	0,83–2,08	1,24	0,78–1,96
Alto	2,8	1,00			

ERI/CET^c^					
Esforço					
Alto	4,6	2,16	1,43–3,26	2,18	1,41–3,36
Baixo	2,1	1,00			
Recompensa					
Baixa	4,0	1,45	0,99–2,14	1,36	0,92–2,02
Alta	2,8	1,00			
Comprometimento excessivo					
Alto	4,5	1,88	1,27–2,78	1,92	1,28–2,88
Baixo	2,4	1,00			

^a^ Riscos relativos ajustados por sexo, idade, escolaridade e turno de trabalho.

^b^ Modelo demanda-controle/apoio social no trabalho.

^c^ Modelo desequilíbrio esforço-recompensa/comprometimento excessivo com o trabalho.

^d^ Valor p < 0,05.

Os modelos parciais (MDC sem AST e ERI sem CET) estiveram associados ao desfecho, exceto em trabalho passivo. Condições de trabalho com alta exigência e desequilíbrio entre esforços e recompensas apresentaram associação com os ATbio nos modelos parciais. Ao incorporar a terceira dimensão ao MDC e ao ERI (modelos completos), houve associação com ATbio, com medidas de maior magnitude, quando comparadas aos modelos parciais. O ERI apresentou magnitude de associação mais forte com ATbio em comparação ao MDC (Tabela 3).

**Tabela 3:** Incidência (%), risco relativo bruto e ajustado de acidentes de trabalho, segundo modelos parciais, completos e combinados de demanda-controle e desequilíbrio esforço-recompensa, em trabalhadores da saúde, Bahia, Brasil, 2012.

Modelo	Acidentes de trabalho				

	I%	RR	IC95%	RRª	IC95%
Modelos parciais					
MDC^b^					
Alta exigência	4,5	1,87	1,06–3,28	1,81	1,01–3,24
Trabalho passivo	3,1	1,29	0,72–2,32	1,51	0,83–2,75
Trabalho ativo	4,5	1,87	1,06–3,29	2,06	1,14–3,71
Baixa exigência	2,4	1,00			
ERI^c^					
Desequilíbrio	5,3	2,24	1,52–3,30	2,10	1,42–3,11
Equilíbrio	2,4	1,00			

Modelos Completos					
MDC/AST^d^					
DC e AST (exposição em ambos)	4,4	2,12	1,10–4,08	1,97	1,03–3,78
Exposição em DC	4,9	2,35	1,05–5,26	2,45	1,10–5,46
Exposição em AST	3,1	1,47	0,74–2,92	1,39	0,70–2,78
Não exposto	2,1	1,00			
ERI/CET^e^					
ERI e CET (exposição em ambos)	5,7	3,01	1,83–4,95	3,01	1,80–5,02
Exposição em ERI	4,3	2,27	1,17–4,38	2,31	1,18–4,50
Exposição em CET	3,4	1,76	1,01–3,08	1,98	1,13–3,46
Não exposto	1,9	1,00			

Modelo Parcial Combinado					
MDC e ERI					
DC e ERI (exposição em ambos)	6,8	3,08	1,88–5,06	2,92	1,78–4,79
Exposição em ERI	3,8	1,74	0,93–3,25	1,71	0,92–3,19
Exposição em DC	2,8	1,25	0,71–2,23	1,39	0,78–2,49
Não exposto	2,2	1,00			

Modelo Completo Combinado					
MDC/AST e ERI/CET					
DC/AST e ERI/CET (exposição em ambos)	8,2	5,83	2,04–16,7	5,23	1,82–15,1
Exposição em DC/AST	2,0	1,42	0,42–4,80	1,79	0,59–5,45
Exposição em ERI/CET	1,3	0,92	0,11–8,13	0,96	0,13–6,80
Não exposto	1,4	1,00			

MDC: modelo demanda-controle; DC: demanda-controle; AST: apoio social no trabalho; ERI: modelo desequilíbrio esforço-recompensa; CET: comprometimento excessivo com o trabalho.

^a^ Riscos relativos ajustados por sexo, idade, escolaridade e turno de trabalho.

^b^ Valor p < 0,05.

Na combinação dos modelos parciais, observou-se associação das dimensões focalizadas com o desfecho. O risco de ATbio foi 2,92 vezes maior entre os expostos em ambos os modelos quando comparado à ausência de exposição nos dois modelos, mesmo após o ajuste por variáveis confundidoras. A frequência de acidentes foi superior quando se analisou o modelo parcial combinado em comparação aos modelos parciais isolados (Tabela 3).

Ao combinar os modelos completos, observou-se maior magnitude da associação com o desfecho em comparação aos demais modelos – parciais e completos isolados e parcial combinado. O risco de ATbio foi 5,23 vezes maior entre os expostos em ambos os modelos completos, em comparação à ausência de exposição nos dois modelos, mesmo após ajuste (Tabela 3).

## DISCUSSÃO

A incidência de acidentes de trabalho com exposição a material biológico foi inferior à encontrada em outros estudos^29^. Ao considerar que a ocorrência do desfecho foi mencionada pelo próprio trabalhador, deve-se considerar a possibilidade de viés de memória e de que os acidentes julgados como menores e sem relevância não tenham sido considerados. Em pesquisa realizada em um hospital universitário, considerar um acidente como de baixo risco já foi apontado como causa de subnotificação de acidentes biológicos por profissionais de enfermagem^32^. Portanto, há de se considerar uma provável subestimativa na análise do resultado encontrado, devido à certa naturalização do risco.

A exposição a alta demanda psicológica mostrou-se associada aos acidentes de trabalho, como observado em outras investigações^6,7,33^. Executar tarefas em ritmo acelerado e em tempo insuficiente pode reduzir a atenção, ampliando a exposição a riscos ocupacionais e a acidentes de trabalho, sobretudo com material biológico no caso dos trabalhadores da saúde.

A dimensão “controle sobre o próprio trabalho”, analisada separadamente, não esteve associada a acidentes. Como o controle sobre o trabalho no grupo estudado (trabalhadores da saúde) é relativamente elevado, em função das demandas do trabalho exigirem uso de habilidades e relativa autoridade decisória, talvez a variabilidade não tenha sido larga o suficiente para apreender possíveis diferenças. A similaridade de escores de uma medida entre grupos definidos dificulta a observação de diferenças significativas. Isso tem sido relatado em pesquisas na área da saúde do trabalhador quando determinadas características do trabalho têm pouca variabilidade interna no grupo ocupacional sob análise. De qualquer modo, quando o baixo controle esteve associado a alta demanda psicológica, aumentaram a exposição a condições estressoras e o efeito sobre a ocorrência de acidentes. Situação de alta exigência (alta demanda e baixo controle simultaneamente) também apresentou associação com afastamento do trabalho devido a acidentes em outros estudos^34^.

O trabalho em saúde é caracterizado pela complexidade das tarefas e pela responsabilidade pelo outro. A atenção do trabalhador pode se dispersar quando as funções são desempenhadas em ritmo intenso, sob pressão do tempo, com sobrecarga de tarefas e vivência frequente de situações imprevistas ou conflitantes que exigem alto nível de conhecimento e de recursos e com o desempenho de múltiplas tarefas^37^. Nesses casos, o estresse afeta a capacidade de concentração, dificultando ações de autocuidado e aumentando o risco de acidentes de trabalho, principalmente quando se trata de tarefas que exigem maior habilidade e destreza, a exemplo do manuseio de perfurocortantes.

Não é raro que o trabalhador, sujeito a condições precárias (recursos humanos e materiais insuficientes), tenha que atender grande número de usuários em um curto espaço de tempo, com demandas diversas, o que o obriga a desenvolver múltiplas e repetitivas tarefas, e nem sempre com a autonomia necessária para a adequada tomada de decisões. Essas situações estressoras, características do trabalho em alta exigência, desgastam o profissional, favorecendo a negligência na realização das tarefas e, consequentemente, aumentando a ocorrência de acidentes de trabalho.

No modelo ERI, a recompensa, que não esteve associada ao desfecho de interesse, refere-se aos ganhos proporcionados pelo trabalho (como salário adequado, respeito e apoio dos colegas, promoções na carreira e estabilidade no emprego). Entretanto, no trabalho em saúde, a recompensa parece estar mais associada a valores simbólicos: o cuidado com o outro e as relações de afeto e vínculo estabelecidas. Assim, os aspectos de recompensa avaliados pelo ERI podem não ter sido adequados o suficiente para detectar essas outras formas de recompensa do trabalho em saúde.

O desequilíbrio esforço-recompensa mostrou-se associado a acidentes de trabalho. Essa desproporção aumentou a incidência de lesões ocupacionais em estudo com trabalhadores coreanos^9^. O trabalhador da saúde lida com a pressão do tempo e da carga pesada de trabalho, intensificada pelas frequentes interrupções e diversas dificuldades enfrentadas durante a jornada. Isso pode gerar ou agravar déficits de concentração na realização das tarefas, o que aumenta o risco de acidentes^37^. Ou seja, não apenas as condições do ambiente de trabalho podem ser inadequadas, mas também as características psicossociais do trabalho que, produzindo situações de tensão e desgaste, de constrangimentos de tempo, de recursos e de desequilíbrio entre esforços e recompensas, podem favorecer a ocorrência de acidentes. Ritmos de trabalho intensos ou volume excessivo de tarefas também podem diminuir a capacidade de atenção e concentração, aumentando a vulnerabilidade do trabalhador. Por isso pesquisas têm reforçado o papel da situação psicossocial nos acidentes de trabalho^5^.

A incorporação de uma terceira dimensão (comprometimento excessivo com o trabalho) ao ERI intensificou a força da associação com ATbio. Considerada um componente intrínseco e subjetivo do modelo, a “superdedicação” envolve altas expectativas de reconhecimento. As pessoas nessa situação costumam se ocupar excessivamente com o trabalho, mostrando dependência do retorno recebido e da aprovação dos outros^13,20^.

Por suas próprias características (estabelecimento de vínculos, cuidado e preocupação com o sofrimento do outro), o trabalho em saúde favorece um envolvimento profissional elevado, que com alguma frequência pode gerar comprometimento excessivo com as atividades desenvolvidas. A vivência do trabalho em saúde como uma vocação, como uma missão de vida, muitas vezes leva o profissional a superestimar o papel do trabalho em relação a outros aspectos da vida^20,38^. Assim, intensas exigências sobre si mesmo são frequentes, impossibilitando o “desligamento” e a vivência de outras situações, o que gera ansiedade e diminui o estado de alerta, consequentemente aumentando a ocorrência de acidentes. Além disso, se a dedicação é muito elevada, a necessidade de recompensas também aumenta, e a frustação dessa expectativa pode produzir ainda mais sofrimento. Areosa e Dwyer^39^ ressaltam a importância de investigar as possíveis pressões presentes no momento do acidente, entre elas a ansiedade, de modo a conhecer melhor os mecanismos que conectam emoções e vivências negativas a acidentes e doenças relacionadas ao trabalho.

Na literatura, o apoio social tem se mostrado importante para atenuar os impactos negativos do estresse ocupacional em trabalhadores da saúde, sendo um fator protetor para a saúde geral^17,20^. Neste estudo, a incorporação do apoio social no trabalho ao MDC contribuiu para melhorar a predição do desfecho, como também observado em outro estudo^17^. O trabalho em saúde é caracterizado por fortes relações interpessoais e colaboração entre colegas. Se há baixo apoio social associado a trabalho de alta exigência, a situação estressora se agrava.

Nos modelos parcial e completo, considerando as medidas de associação pontuais, o ERI mostrou capacidade de identificar situações de exposição mais sensíveis do que o MDC. O modelo de Karasek^12^ é amplamente utilizado na literatura para investigar a associação entre estressores no trabalho e os mais variados desfechos. Ele foi proposto na década de 1970 para avaliar o trabalho industrial, com destaque para os aspectos envolvidos na realização das tarefas, o que traz dificuldades para sua aplicação no trabalho em saúde. As características específicas desse trabalho, baseado em complexas relações humanas, não permitem a mera transposição do modelo fundamentado no processo industrial de produção^14^. Assim, Söderfeldt et al.^40^ apontam a necessidade de adequar dimensões do MDC de modo a possibilitar seu uso nas organizações de serviços humanos. O ERI, ao incorporar a recompensa no trabalho, pode ter aumentado o poder explicativo do modelo em relação às condições do trabalho na saúde em comparação ao MDC.

O uso combinado dos modelos do MDC e ERI aumentou o poder preditivo da relação entre estressores e acidentes de trabalho. Outros estudos também têm evidenciado melhor desempenho na identificação de situações desfavoráveis à saúde com a combinação de modelos, que parece ser uma alternativa promissora às limitações do uso de modelos isolados para abarcar a complexidade do ambiente psicossocial do trabalho^18,20^.

Esses modelos abordam aspectos específicos do trabalho (o MDC avalia elementos relativos à tarefa, às demandas e ao grau de autonomia envolvidos, enquanto o ERI volta-se a questões de injustiça, de desequilíbrio nas trocas no trabalho), mas, quando utilizados conjuntamente, permitem avaliar de modo mais amplo as características e os efeitos de diferentes fatores estressores presentes simultaneamente. Os dados obtidos reforçam a relevância desse emprego combinado dos modelos, que possibilita uma compreensão mais abrangente dos aspectos psicossociais do trabalho. Griep et al.^41^ observaram que diferentes combinações de estressores resultavam em predições diferentes de absenteísmo. Embora os modelos parciais tenham mostrado bom desempenho para identificar situações de risco para ATbio, a combinação das dimensões abordadas nos dois modelos testados permitiu identificar situações de maior exposição, que merecem maior atenção na prevenção de ATbio. Identificadas essas situações e os grupos que podem estar expostos, pode-se reorganizar o trabalho e as dimensões aqui analisadas, incorporando ações, por exemplo, aos programas de biossegurança.

Algumas limitações do presente estudo devem ser destacadas. Medidas autorreferidas, como já mencionado, podem sofrer influência de viés de memória. Além disso, devido à investigação ter sido conduzida com trabalhadores que estavam em pleno exercício de suas atividades laborais, não se pode desconsiderar o efeito do trabalhador sadio^42^. Assim, podem não ter participado da pesquisa os profissionais afastados em decorrência de acidentes de trabalho ou de outros agravos à saúde de origem ocupacional.

Apesar dessas limitações potenciais, os resultados obtidos reforçam a relevância dos aspectos psicossociais do trabalho na exposição a ATbio. Evidenciou-se ainda o aumento do potencial explicativo para os ATbio quando se emprega os modelos parciais e completos combinadamente, sendo este um importante achado deste estudo. A análise de várias dimensões do trabalho ampliou a compreensão das conexões que geram situações de vulnerabilidade aos ATbio, com análise detalhada daquelas relacionadas à estrutura psicossocial do trabalho.

Conclui-se que, devido às diferentes dimensões abordadas pelos modelos empregados (as quais se complementam e permitem identificar mais detalhadamente as exposições ocupacionais), seu uso combinado pode subsidiar intervenções mais efetivas nos ambientes e processos do trabalho em saúde, visando à prevenção dos ATbio e de outros agravos não abordados no presente estudo. Ou seja, os resultados evidenciam a necessidade de programas de prevenção de ATbio que não se limitem apenas ao controle de aspectos de biossegurança, mas incorporem também aspectos psicossociais do trabalho de modo mais amplo possível.
